# An Efficient Multiplex PCR-Based Assay as a Novel Tool for Accurate Inter-Serovar Discrimination of *Salmonella* Enteritidis, *S*. Pullorum/Gallinarum and *S*. Dublin

**DOI:** 10.3389/fmicb.2017.00420

**Published:** 2017-03-16

**Authors:** Dan Xiong, Li Song, Jing Tao, Huijuan Zheng, Zihao Zhou, Shizhong Geng, Zhiming Pan, Xinan Jiao

**Affiliations:** ^1^Jiangsu Key Laboratory of Zoonosis, Yangzhou UniversityYangzhou, China; ^2^Jiangsu Co-innovation Center for Prevention and Control of Important Animal Infectious Diseases and ZoonosesYangzhou, China; ^3^Joint International Research Laboratory of Agriculture and Agri-product Safety of the Ministry of EducationYangzhou, China; ^4^Key Laboratory of Prevention and Control of Biological Hazard Factors (Animal Origin) for Agrifood Safety and Quality, Ministry of Agriculture of China, Yangzhou UniversityYangzhou, China

**Keywords:** *Salmonella* Enteritidis, *Salmonella* Pullorum/Gallinarum, *Salmonella* Dublin, multiplex PCR, accurate discrimination

## Abstract

*Salmonella enterica* serovars Enteritidis, Pullorum/Gallinarum, and Dublin are infectious pathogens causing serious problems for pig, chicken, and cattle production, respectively. Traditional serotyping for *Salmonella* is costly and labor-intensive. Here, we established a rapid multiplex PCR method to simultaneously identify three prevalent *Salmonella* serovars Enteritidis, Pullorum/Gallinarum, and Dublin individually for the first time. The multiplex PCR-based assay focuses on three genes *tcpS, lygD*, and *flhB*. Gene *tcpS* exists only in the three *Salmonella* serovars, and *lygD* exists only in *S*. Enteritidis, while a truncated region of *flhB* gene is only found in *S*. Pullorum/Gallinarum. The sensitivity and specificity of the multiplex PCR assay using three pairs of specific primers for these genes were evaluated. The results showed that this multiplex PCR method could accurately identify *Salmonella* Enteritidis, Pullorum/Gallinarum, and Dublin from eight non-*Salmonella* species and 27 *Salmonella* serovars. The least concentration of genomic DNA that could be detected was 58.5 pg/μL and the least number of cells was 100 CFU. Subsequently, this developed method was used to analyze clinical *Salmonella* isolates from one pig farm, one chicken farm, and one cattle farm. The results showed that blinded PCR testing of *Salmonella* isolates from the three farms were in concordance with the traditional serotyping tests, indicating the newly developed multiplex PCR system could be used as a novel tool to accurately distinguish the three specific *Salmonella* serovars individually, which is useful, especially in high-throughput screening.

## Introduction

*Salmonella* is a prominent food-borne pathogen, capable of causing serious illness in humans, including gastroenteritis, typhoid fever, septicemia, and sometimes even death (Tatavarthy and Cannons, 2010). It is reported almost 75% of *Salmonella* infections in human cases are caused by contaminated food products, including pork, poultry, and beef (Hald et al., 2004).

Although more than 2,600 *Salmonella* serovars exist (Ranieri et al., 2013; Xiong et al., 2016), *S*. Enteritidis, *S*. Pullorum/Gallinarum, and *S*. Dublin are the main serovars causing animal diseases (Nielsen, 2013; Saeki et al., 2013; Zhu et al., 2015). *S*. Enteritidis could cause severe infection in humans (Rodrigue et al., 1990; Nesbitt et al., 2012), and was the main serovar in the contaminated food products and infected individuals in southern Brazil between 1999 and 2008 (Paião et al., 2013). *S*. Gallinarum only infects birds and has two biovars Gallinarum and Pullorum, causing fowl typhoid and “white diarrhea,” respectively (Soria et al., 2012; Xiong et al., 2016). Particularly, *S*. Gallinarum could transmit to the reproductive system and result in salmonellosis (Keller et al., 1997). *S*. Dublin causes widespread losses in cattle husbandry, mainly as a result of increased levels of abortion, mortality, and morbidity, and a reduced milk yield, and has attracted considerable attention from cattle industries worldwide (Carrique-Mas et al., 2010; Nielsen and Dohoo, 2013). Human infections are most caused by the consumption of milk or beef products (Nielsen, 2013). Thus, timely detection of the three prominent *Salmonella* serovars, *S*. Enteritidis, *S*. Pullorum/Gallinarum, and *S*. Dublin, is very essential and urgent.

Traditional serotyping for *Salmonella* is based on the identification of the somatic (O) and flagellar (H) antigens by using specific sera following the White-Kauffmann-Le Minor scheme (Majchrzak et al., 2014). Many useful data could be obtained by *Salmonella* serotyping. Thus, an accurate diagnostic method for *Salmonella* serovars is highly important for public health. Despite its wide use, traditional *Salmonella* serotyping has many disadvantages, which is expensive, time-consuming and labor-intensive (Ranieri et al., 2013). Recent studies showed that polymerase chain reaction (PCR) can be a useful method to detect pathogens for its high specificity and sensitivity (Abdissa et al., 2006; Moyo et al., 2007). PCR-based method for *Salmonella* serotyping is a rapid and economical tool (Karns et al., 2015). Gene *lygD* in Sdf locus has been found only in *S*. Enteritidis and could be used to distinguish this serovar specifically (Zhu et al., 2015). Previously, we have proved that *flhB* gene can be used to detect *S*. Pullorum/Gallinarum because a unique region was deficient only in this serovar (Xiong et al., 2016).

In the present study, we established a rapid multiplex PCR method to distinguish the three prevalent *Salmonella* serovars Enteritidis, Pullorum/Gallinarum, and Dublin individually for the first time. The approach was based on designing three pairs of primers targeting *tcpS, lygD*, and *flhB* genes. The sensitivity and specificity of the multiplex PCR assay were determined, and the PCR assay was used to detect three sets of *Salmonella* isolates from one pig farm, one chicken farm, and one cattle farm. The newly developed multiplex PCR with three pairs of primers could be used as a novel tool to timely identify the three specific *Salmonella* serovars, and reinforce the traditional *Salmonella* serotyping method, particularly in high-throughput screening.

## Materials and methods

### Bacterial strains

Strains of *Salmonella* and non-*Salmonella* organisms, including *S*. Enteritidis, *S*. Gallinarum, *S*. Pullorum, *S*. Dublin, *S*. Meleagridis, *S*. Uganda, *S*. Anatis, *S*. London, *S*. Rissen, *S*. Typhimurium, *S*. Derby, *S*. Choleraesuis, *S*. Sinstorf, *S*. Indiana, *S*. Newlands, *S*. Dumfries, *S*. Muenster, *S*. Yoruba, *S*. Kentucky, *S*. Agona, *S*. Senftenberg, *S*. Thompson, *S*. Blockley, *S*. Inchpark, *S*. Farsta, *S*. Dabou, *S*. Virchow, *Mycobacterium tuberculosis, Brucella abortus, Listeria monocytogenes, Campylobacter jejuni*, and *Escherichia coli*, were commercially available or previously isolated in our routine monitoring (Table 1).

**Table 1:** *****Salmonella*** and non-***Salmonella*** strains used to evaluate the specificity and sensitivity of the developed multiplex PCR method**.

	**Strain**	**Serovar/species**	**Source**	**Multiplex PCR results**		
				***tcpS***	***lygD***	***flhBinner***
*Salmonella*	C50041	Enteritidis	Laboratory stock	+	+	+
	C50336	Enteritidis	Laboratory stock	+	+	+
	S06004	Pullorum	Laboratory stock	+	−	−
	6508	Pullorum	Isolate from chicken	+	−	−
	SG9	Gallinarum	Wigley et al., 2005	+	−	−
	SL5928	Dublin	Laboratory stock	+	−	+
	T3	Uganda	Cai et al., 2016	−	−	+
	T9	Meleagridis	Li et al., 2016	−	−	+
	T8	Anatis	Li et al., 2016	−	−	+
	G2	London	Cai et al., 2016	−	–	+
	ZX	Rissen	Cai et al., 2016	−	−	+
	Y7	Derby	Cai et al., 2016	−	−	+
	Y8	Typhimurium	Li et al., 2016	−	−	+
	C500	Choleraesuis	Laboratory stock	−	−	+
	ZH65	Indiana	Cai et al., 2016	−	−	+
	ZH5	Sinstorf	Laboratory stock	−	−	+
	ZH10	Newlands	Isolate from cattle	−	−	+
	ZH24	Muenster	Laboratory stock	−	−	+
	ZH82	Yoruba	Isolate from pig	−	−	+
	G449	Dumfries	Laboratory stock	−	−	+
	G241	Kentucky	Laboratory stock	−	−	+
	G382	Agona	Laboratory stock	−	−	+
	ZH35	Thompson	Cai et al., 2016	−	−	+
	P192	Senftenberg	Laboratory stock	−	−	+
	G439	Blockley	Laboratory stock	−	−	+
	G86	Inchpark	Laboratory stock	−	−	+
	P122	Virchow	Laboratory stock	−	−	+
	P74	Farsta	Laboratory stock	−	−	+
	G85	Dabou	Laboratory stock	−	−	+
Non-*Salmonella*	H37Rv	*Mycobacterium tuberculosis*	ATCC 27294	−	−	−
	11168	*Campylobacter jejuni*	ATCC 700819	−	−	−
	110	*Campylobacter jejuni*	Isolate from chicken	−	−	−
	S19	*Brucella abortus*	Laboratory stock	−	−	−
	EGDe	*Listeria monocytogenes*	ATCC BAA-679	−	−	−
	JS15	*Listeria monocytogenes*	Isolate from sheep	−	−	−
	1314	*Escherichia coli*	Isolate from chicken	−	−	−
	1352	*Escherichia coli*	Isolate from chicken	−	−	−

### Bacterial growth and genomic DNA isolation

The bacterial culture and DNA isolation were performed as previously described (Xiong et al., 2016). Briefly, all strains used in the study were inoculated in Brain Heart Infusion broth (Becton, Dickinson and Company, Sparks, MD, USA) or Luria-Bertani broth (Oxoid, Basingstoke, Hampshire, England) at 37°C overnight with an agitation speed of 180 rpm. Bacterial DNA was harvested with a TIANamp Bacterial DNA kit (TianGen, Beijing, China). The purity and concentration of the extracted DNA were determined using a NanoDrop ND-1000 (Thermo Scientific, Wilmington, DE, USA), and DNA samples were stored at −20°C until use.

### *In silico* analysis

To establish a sequence- and PCR-based *Salmonella* serotyping method for discrimination of *S*. Enteritidis, *S*. Pullorum/Gallinarum, and *S*. Dublin individually, the basic local alignment search tool (BLAST) algorithm (NCBI, non-redundant nucleotide collection) was applied. The *tcpS, lygD*, and *flhB* nucleotide sequences were used against the nucleotide collection (nr/nt) database, respectively. The aligned sequence number was set to the maximal value 20,000 with other parameters set to the default values. The three pairs of primers for the targets were designed using Primer Premier 5 (Premier, Palo Alto, CA, USA).

### PCR procedure

PCRs were conducted in a 25 μL reaction volume, consisting of 100 ng of genomic DNA template, 1 × polymerase buffer, 1 U of *Taq* polymerase (Takara Biotechnology Co., Dalian, China), 200 μM of deoxynucleoside triphosphate, and 80 nM of *tcpS/lygD/flhBinner* primers. PCR were conducted with a T100 Thermal Cycler (Bio-Rad, Hercules, California, USA) as follows: 94°C for 5 min, 30 cycles of 94°C for 45 s, 55°C for 45 s, and 72°C for 1 min, followed by 72°C for 10 min. The amplified PCR products were analyzed on the 1% agarose gel in 1 × TAE buffer.

### Specificity of the multiplex PCR

The specificity of the three pairs of primers in the multiplex PCR system was evaluated by detecting genomic DNA from 29 different *Salmonella* strains, which included 27 *Salmonella* serovars and eight non-*Salmonella* species (Table 1).

### Sensitivity of the multiplex PCR

The genomic DNA of *S*. Enteritidis strain C50041, *S*. Pullorum strain S06004 and *S*. Dublin strain SL5928 were 10-fold continuously diluted from 58.5 ng/μL to 585 fg/μL, respectively. Each dilution (2 μL) was used in the multiplex PCR assay. This assay was to determine the minimum limit of DNA that can be detected by the multiplex PCR method.

*S*. Enteritidis strain C50041, *S*. Pullorum strain S06004, and *S*. Dublin strain SL5928 were cultured overnight and the bacterial concentration was evaluated by the plate count assay. The bacterial culture was washed with phosphate buffered saline (PBS) twice, 10-fold serially diluted to the concentrations 2 × 10^7^ to 2 × 10^2^ CFU/mL, and boiled for 10 min to collect the genomic DNA. In the multiplex PCR method, each dilution (5 μL) was used as templets to determine the minimum cells of *Salmonella* that can be detected.

### Isolation of *Salmonella* strains from different farms

Additional clinical *Salmonella* strains with unknown serovars were obtained from three farms, one pig farm, one chicken farm, and one cattle farm in Yangzhou, China. The *Salmonella* isolates were collected from feces, floors and lairage, and identified as previously described methods (Cai et al., 2016; Li et al., 2016; Xiong et al., 2016). In brief, each sample was suspended in 50 mL buffered peptone water (Difco, BD, Sparks, MD, USA) and followed by incubation at 37°C for 16–18 h. This broth culture (0.1 mL) was subcultured in 10 mL of Rappaport–Vassiliadis enrichment broth (Difco, BD) at 42°C for 24 h. After incubation, the RV broth was streaked onto xylose lysine tergitol 4 (Difco, BD), and cultured at 37°C for 24–48 h. The presumptive *Salmonella* colonies were picked from all plates and followed by biochemically confirmation using an API-20E test kit (bioMérieux, Marcy l'Etoile, France).

### Application of the multiplex PCR method on clinical samples

The multiplex PCR method was applied to detect the genomic DNA of *Salmonella* isolates from one pig farm (24 *Salmonella* isolates), one chicken farm (24 *Salmonella* isolates), and one cattle farm (11 *Salmonella* isolates). The obtained results of the developed PCR method were compared with traditional *Salmonella* serotyping approach.

### Traditional serotyping of *Salmonella* isolates from different farms

The traditional serotyping for all isolated *Salmonella* strains from the pig, chicken and cattle farms were conducted by slide agglutination assay using the specific antisera (Tianrun Bio-Pharmaceutical, Ningbo, China) following the White-Kauffmann-LeMinor instructions (Grimont and Weill, 2007).

## Results

### *In silico* analysis and primer design

*In silico* analysis showed that *tcpS* exists only in *S*. Enteritidis, *S*. Pullorum/Gallinarum, and *S*. Dublin, and has 98–100% sequence similarity across the three *Salmonella* serovars in the database. Although *tcpS* in one *E. coli* strain showed 89% DNA sequence identity to the *Salmonella tcpS*, it does not contain the C-terminal region of *tcpS* or a match to the *tcpS*-R primer site (data not shown). *lygD* gene exists only in *S*. Enteritidis, and shares 98–100% sequence similarity among this serovar in the database (data not shown). Our previous study showed that *flhB* gene of *S*. Pullorum/Gallinarum lacks a unique region *flhBinner* compared with other serovars, and could be used to identify *S*. Pullorum/Gallinarum (Xiong et al., 2016). Therefore, three pairs of oligonucleotide primers distinguishing three specific *Salmonella* serovars were designed based on the three targets *tcpS, lygD* and *flhBinner* (Table 2).

**Table 2:** **Multiplex PCR primers used for identification of *Salmonella* Enteritidis, *S*. Pullorum/Gallinarum, and *S*. Dublin**.

**Primers**	**Primer sequence (5′ → 3′)**	**Size (bp)**	**Accession no./Nt segments**	***Salmonella* serovars**		
				**SE**	**SP/SG**	***SD***
*tcpS* F	ATGTCTATAAGCACCACAATG	882	KM408432.1 1–882	+	+	+
*tcpS* R	TCATTTCAATAATGATTCAAGC					
*lygD* F	CATTCTGACCTTTAAGCCGGTCAATGAG	339	CP007175.1 1468298–1468636	+	−	−
*lygD* R	CCAAAAAGCGAGACCTCAAACTTACTCAG					
*flhBinner* F	GCGGACGTCATTGTCACTAACCCGACG	155	CP014983.1 2041558–2041712	+	−	+
*flhBinner* R	TCTAAAGTGGGAACCCGATGTTCAGCG					

*SE, S. Enteritidis; SP/SG, S. Pullorum/Gallinarum; SD, S. Dublin*.

### Specificity of the multiplex PCR assay

**Figure 1:**
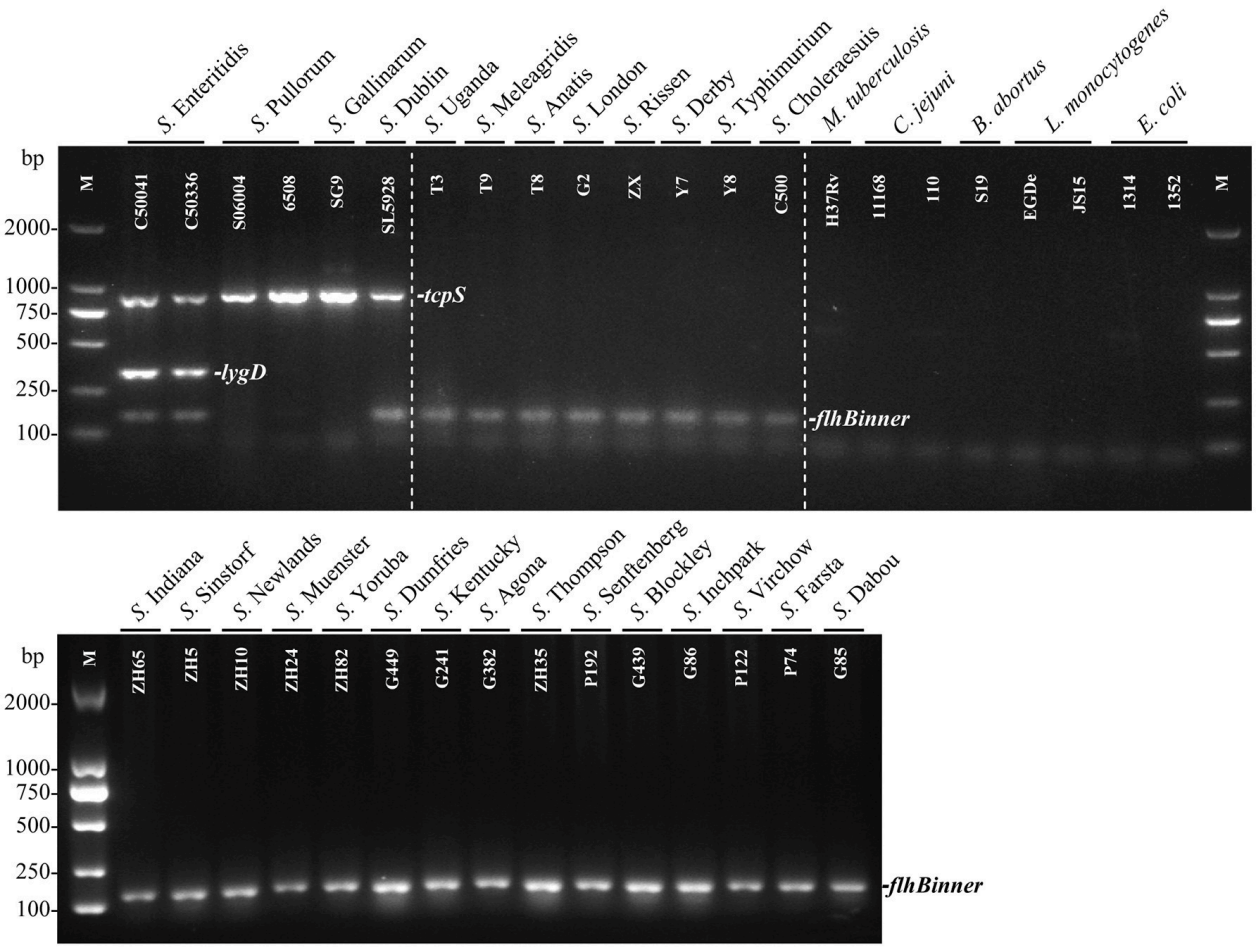
**Specificity of the multiplex PCR method for the identification of *Salmonella* serovars Enteritidis, Pullorum/Gallinarum, and Dublin**. The multiplex PCR assays, using genomic DNA from various *Salmonella* and non-*Salmonella* strains, were conducted using the designed primers targeting *tcpS* (882 bp), *lygD* (339 bp), and *flhBinner* (155 bp). The three specific PCR products could be amplified in *S*. Enteritidis. *tcpS* and *flhBinner* could be amplified in *S*. Dublin, while only *tcpS* gene could be amplified in *S*. Pullorum/Gallinarum. Detailed strain information is given in Table 1.

The specificity of the multiplex PCR method was determined by detecting 29 *Salmonella* strains and eight non-*Salmonella* species. The results showed that *S*. Enteritidis generated three specific bands for *tcpS, lygD* and *flhBinner*, and *S*. Dublin generated two specific bands for *tcpS* and *flhBinner*, while *S*. Pullorum/Gallinarum generated only one specific band for *tcpS*. In contrast, only one band of *flhBinner* was generated in the other 23 *Salmonella* serovars, and no amplification product was observed in all non-*Salmonella* pathogens (Figure 1).

### Sensitivity of the multiplex PCR assay

**Figure 2:**
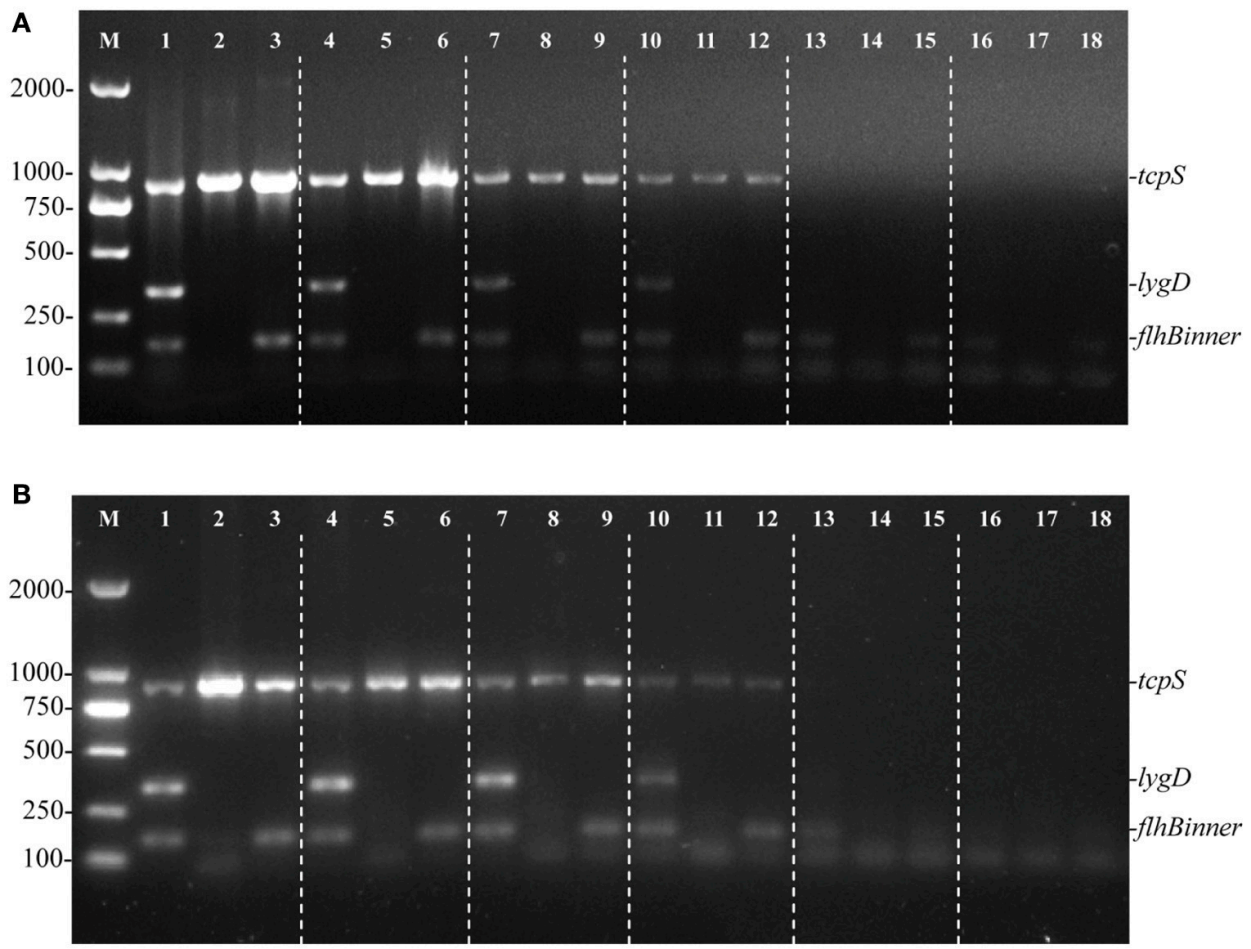
**Sensitivity of the multiplex PCR method for the detection of genomic DNA and cells from *S*. Enteritidis (C50041), *S*. Pullorum (S06004), and *S*. Dublin (SL5928)**. The multiplex PCR amplifies three specific bands of *tcpS* (882 bp), *lygD* (339 bp), and *flhBinner* (155 bp). Lane M: DL2000 DNA marker (Takara Biotechnology Co., Dalian, China). The multiplex PCR for the detection of genomic DNA **(A)** and *Salmonella* cells **(B)**, lanes 1, 4, 7, 10, 13, 16 (*S*. Enteritidis), 2, 5, 8, 11, 14, 17 (*S*. Pullorum), and 3, 6, 9, 12, 15, 18 (*S*. Dublin): Genomic DNA used as templates at the following concentrations, respectively: 58.5 ng/μL, 5.85 ng/μL, 585 pg/μL, 58.5 pg/μL, 5.85 pg/μL, 585 fg/μL; the number of cells per PCR assay at the following concentrations, respectively: 10^5^, 10^4^, 10^3^, 10^2^, 10^1^, and 10^0^ CFU.

To evaluate the sensitivity of the multiplex PCR method, genomic DNA of *S*. Enteritidis, Pullorum, and Dublin were consecutively diluted and used as templates. The targeted fragments could be amplified at the lowest concentration of 58.5 pg/μL (Figure 2A), suggesting 58.5 pg/μL of genomic DNA was needed to detect *S*. Enteritidis, Pullorum, or Dublin using this method. Besides, the minimum cells of *S*. Enteritidis, Pullorum, and Dublin that could be detected using this multiplex PCR method were determined. After detecting various dilutions of *Salmonella* cells, we validated that the least cells that could be detected was 100 CFU (Figure 2B).

### Application of the multiplex PCR method

To determine the effectiveness of the developed multiplex PCR method, samples from one pig farm (24 *Salmonella* isolates), one chicken farm (24 *Salmonella* isolates), and one cattle farm (11 *Salmonella* isolates) were examined. The PCR results showed that three isolates from the pig farm generated three specific bands of *tcpS, lygD*, and *flhBinner*, suggesting that the three isolates were *S*. Enteritidis. Five samples from the chicken farm generated three specific bands of *tcpS, lygD*, and *flhBinner*, and 11 samples generated only one specific band of *tcpS*, suggesting that the five isolates and the other 11 isolates were *S*. Enteritidis and *S*. Pullorum/Gallinarum, respectively. Among the isolates from the cattle farm, only one sample generated two specific bands of *tcpS* and *flhBinner*, suggesting that this isolate were *S*. Dublin (Table 3).

**Table 3:** *****Salmonella*** strains isolated from three different farms to examine the application of the developed multiplex PCR method**.

**Source**	**Serovar (no. of isolates)**	**Isolate no**.	**PCR results**			**Source**	**Serovar (no. of isolates)**	**Isolate no**.	**PCR results**		
			***tcpS***	***lygD***	***flhB inner***				***tcpS***	***lygD***	***flhB inner***
Pig farm	Enteritidis (3)	Pi9	+	+	+			Ch14	+	−	−
		Pi17	+	+	+			Ch16	+	−	−
		Pi21	+	+	+			Ch18	+	−	−
	Derby (9)	Pi1	−	−	+			Ch20	+	−	−
		Pi2	−	−	+			Ch21	+	−	−
		Pi5	−	−	+		Enteritidis (5)	Ch6	+	+	+
		Pi7	−	−	+			Ch8	+	+	+
		Pi12	−	−	+			Ch17	+	+	+
		Pi13	−	–	+			Ch22	+	+	+
		Pi18	–	−	+			Ch24	+	+	+
		Pi22	−	−	+		Indiana (5)	Ch1	−	−	+
		Pi23	−	–	+			Ch4	−	−	+
	Typhimurium (5)	Pi3	–	−	+			Ch9	−	−	+
		Pi10	−	−	+			Ch11	−	−	+
		Pi14	−	−	+			Ch23	−	−	+
		Pi20	−	−	+		Thompson (3)	Ch2	−	−	+
		Pi24	−	–	+			Ch15	–	–	+
	London (2)	Pi8	–	−	+			Ch19	−	−	+
		Pi16	−	−	+	Cattle farm	Dublin (1)	Ca7	+	−	+
	Rissen (5)	Pi4	−	−	+		Newlands (8)	Ca1	−	−	+
		Pi6	−	−	+			Ca2	−	−	+
		Pi11	−	−	+			Ca3	−	−	+
		Pi15	−	−	+			Ca5	−	−	+
		Pi19	−	−	+			Ca6	−	−	+
Chicken farm	Pullorum (11)	Ch3	+	−	−			Ca8	−	−	+
		Ch5	+	−	−			Ca9	−	−	+
		Ch7	+	–	–			Ca11	–	–	+
		Ch10	+	−	−		Muenster (2)	Ca4	−	−	+
		Ch12	+	−	−			Ca10	−	−	+
		Ch13	+	−	−						

*The serotyping of the Salmonella isolates was determined based on the traditional serotyping tests according to the White-Kauffmann-Le Minor scheme*.

### Traditional serotyping of *Salmonella* isolates

The serotypes of *Salmonella* isolates from the three farms were identified using slide agglutination assays using specific O and H antisera. The isolates from the pig farm were obtained from lairage, floors, and feces, and included three strains of *S*. Enteritidis, nine strains of *S*. Derby, five strains of *S*. Typhimurium, two strains of *S*. London, and five strains of *S*. Rissen. The isolates from the chicken farm were obtained from fecal samples and floors, and included 11 strains of *S*. Pullorum, five strain of *S*. Enteritidis, five strains of *S*. Indiana, and three strains of *S*. Thompson. The isolates from the cattle farm were obtained from fecal samples, and included one strain of *S*. Dublin, eight strains of *S*. Newlands, and two strains of *S*. Muenster. The traditional serotyping results showed complete concordance with the developed multiplex PCR methods for all samples (Table 3).

## Discussion

*Salmonella* remains the most frequently isolated bacteria among food-borne pathogens, and over 19,000 cases were reported in the USA in 2013 (Crim et al., 2014). Thus, a simple method to detect and monitor *Salmonella* serovars in farms is urgently required. Several approaches based on antigens and DNA analysis have been developed to detect *Salmonella* in foodstuffs, including enzyme-linked immunosorbent assay, PCR analysis, and next generation sequencing (Ricke et al., 2013; Park et al., 2014).

Traditional serotyping could provide subtyping information that allows worldwide comparison. This has promoted the characterization of many international *Salmonella* outbreaks (Werber et al., 2005; Elviss et al., 2009). Furthermore, comparison with historical data was also available based on serotyping because of its wide use for almost 70 years. Verifying the causative pathogens is usually the essential first step in many important epidemiological investigations. Traditional serotyping could be a tough task because it requires necessary expertise and numerous antisera to interpret the agglutination results (Hong et al., 2008). Traditional serotyping methods are also labor-intensive, complicated, expensive, and time-consuming. More importantly, morphological descriptions and biochemical tests may produce ambiguous results (de Freitas et al., 2010). Although whole genome sequencing is becoming more accessible and has been used as a genoytping method, it could be costly and time-consuming, and not practical for sequencing numerous isolates simultaneously. Therefore, rapid PCR-based detection systems for *Salmonella* have been developed in recent years (Persson et al., 2012).

Comparative genomic analysis is becoming common to validate novel serovar-specific genes because of the improved BLAST program and continuously supplemented genomic data (Zhai et al., 2014). This approach is more economical, convenient, and effective than traditional methods. For example, serovar-specific sequences (STM4495 and SEN1392) for identifying *S*. Enteritidis and *S*. Typhimurium were obtained by comparative genomics (Liu et al., 2012). At present, *vagC*, located in the *Salmonella* virulence plasmid, is considered a better target for PCR detection of *S*. Dublin (Persson et al., 2012). However, false-positive results still occur, such as misidentification of a *S*. Muenchen serovar as *S*. Dublin (Zhai et al., 2014). Previously, we have found *Salmonella flhB* gene could be used to identify *S*. Pullorum/Gallinarum from others because a unique region was deficient only in this serovar (Xiong et al., 2016). Here, we took advantage of three *Salmonella* genes, *tcpS, lygD*, and *flhB*, which were predicted by comparative genomic analysis, to design primers that can accurately distinguish *Salmonella* serovars Enteritidis, Pullorum/Gallinarum, and Dublin. This allowed the development of a reliable and rapid multiplex PCR method to screen these three serovars individually. To the best of our knowledge, it is the first one-step multiplex PCR method to detect these three prominent *Salmonella* serovars individually.

The multiplex PCR method produced positive results in *S*. Enteritidis, *S*. Pullorum/Gallinarum, and *S*. Dublin only, with negative results obtained in other *Salmonella* serovars and eight non-*Salmonella* bacteria (Figure 1). Besides, the PCR method is very rapid and takes about 3 h to complete. Thus, the PCR results agreed with the BLAST analysis, and the proposed application of the multiplex PCR method was verified by screening the three prominent *Salmonella* serovars in samples isolated from pig, chicken, and cattle farms. The results described in this study provide a proof of concept and demonstrate the feasibility of using this PCR method to screen *S*. Enteritidis, Pullorum/Gallinarum, and Dublin. Future studies will investigate different approaches to isolate DNA directly from infected animals and determine if it can be applied in the field.

This multiplex PCR method could be used for a rapid screening of the three specific *Salmonella* serovars and simplify the procedures of traditional serotyping. Besides, the combination of traditional serotyping methods and the developed PCR-based approach would promote the efficiency for the serovar identification of *Salmonella* strains.

## Conclusion

In summary, an efficient multiplex PCR method targeting three prominent *Salmonella* serovars, *S*. Enteritidis, *S*. Pullorum/Gallinarum, and *S*. Dublin, was identified for the first time. The multiplex PCR method was based on three genes of *tcpS, lygD*, and *flhB*, and the specificity and sensitivity of the multiplex PCR method were determined. The multiplex PCR system was exploited to test extensive sets of *Salmonella* strains isolated from three farms, thus validating the effectiveness and specificity of the assay. The results suggest that the developed rapid and efficient multiplex PCR assay could be used as a novel and high-throughput diagnostic tool for simultaneous identification of the three specific *Salmonella* serovars.

## Author contributions

ZP and XJ designed the experiments; DX and LS performed the PCR assays; DX, JT, HZ, and ZZ isolated the samples from the chicken farm; SG participated in the data analysis and interpretation; DX, ZP, and XJ wrote the paper. All authors read and approved the final manuscript.

### Conflict of interest statement

The authors declare that the research was conducted in the absence of any commercial or financial relationships that could be construed as a potential conflict of interest.
